# The effects of plyometric training on athletic’ jump performance: a systematic review and meta-analysis 

**DOI:** 10.3389/fphys.2025.1683281

**Published:** 2025-10-24

**Authors:** Shuzhen Ma, Diliang Wang, Kim Geok Soh, Xinzhi Wang, Yanqi Xu, Qiang Tu

**Affiliations:** ^1^ School of Public Administration, Guilin University of Technology, Guilin, China; ^2^ Department of Sports Studies, Faculty of Educational Studies, Universiti Putra Malaysia, Selangor, Malaysia; ^3^ School of Physical Education and Health, Guilin Institute of Information Technology, Guilin, China; ^4^ College of Materials Science and Engineering, Guilin University of Technology, Guilin, China; ^5^ Faculty of Medicine and Health, The University of Sydney, Sydney, NSW, Australia

**Keywords:** plyometric training, jump performance, healthy athletes, a systematic review, meta-analysis

## Abstract

**Background:**

Jump performance is essential in sports and relies on explosive power. Plyometric training (PT) specifically targets explosive power, while routine training (RT) focuses on general strength or sport-specific skills. Although many studies have shown that PT improves jump performance, few have directly compared PT and RT across different jump types. This study aims to address this gap.

**Objective:**

This study compares the effectiveness of plyometric and regular training on enhancing jump performance in healthy athletes.

**Method:**

Five electronic databases (PubMed, ERIC, Web of Science, EBSCOhost, and Scopus) were comprehensively searched for relevant studies. Review Manager software was used for statistical analyses. The quality of included studies and risk of bias were assessed using the Cochrane Risk of Bias tool by two independent reviewers, with disagreements resolved by consensus. Forest plots and subgroup analyses were conducted for metrics with a sample size of three or more. Heterogeneity was assessed by using the I^2^ statistic (25% for low, 50% for moderate, and 75% for high). A random-effects model was used for high heterogeneity and a fixed-effects model for low heterogeneity. Estimates were reported with confidence intervals, and significance was set at α = 0.05.

**Results:**

The meta-analysis showed that plyometric training significantly improved counter movement jump performance compared to routine training (SMD = 1.99, 95% CI [1.50, 2.48], p < 0.001, I^2^ = 0%). For squat jump, there was no significant difference between the two training methods (SMD = 0.96, 95% CI [-0.10, 2.02], p = 0.07, I^2^ = 0%). Vertical jump performance also showed no significant difference (SMD = 2.90, 95% CI [-0.50, 6.30], p = 0.09, I^2^ = 63%). Subgroup analysis of vertical jump performance indicated low heterogeneity for female subjects (I^2^ = 0%) and high heterogeneity for male subjects (I^2^ = 71%), primarily due to the inclusion of one outlier study. Excluding this study reduced the heterogeneity to 5% (SMD = 3.20, 95% CI [1.80, 4.60], p < 0.001).

**Conclusion:**

This study shows that plyometric training significantly improves counter movement jump performance compared to routine training. However, there is no significant difference between the two training methods in squat jump and vertical jump performance. This study demonstrates that plyometric training significantly enhances counter movement jump performance, while showing no superior effects on squat jump and vertical jump performance compared with routine training.

**Systematic Review Registration:**

https://www.10.37766/inplasy2024.6.0107.

## 1 Introduction

Plyometric training is a fast, explosive exercise that includes a pre-lengthening or reversing movement and a complete stretch-shortening cycle (SSC) (Wilk et al., 1993). It is a form of strength training primarily consisting of various types of jumps (Sole et al., 2022). The SSC involves an eccentric contraction immediately followed by a concentric contraction, which enhances the storage of elastic energy and neuromuscular efficiency, thereby improving overall performance (Komi, 2000; Markovic, 2007; Farentinos and Radcliffe, 1999). The mechanisms of plyometric training mainly include three parts: the first part converts the elastic energy stored during muscle stretching into output power during concentric contraction (Wilk et al., 1993); the second part utilizes the muscle stretching process to engage the stretch reflex (Bal et al., 2012); and the third part sends sensory signals to the spinal cord, transmitting information to α-motoneurons, and inhibiting the contraction of antagonist muscles (Potach, 2004). Plyometric training has the potential and training advantages in optimizing SSC and related neuromechanical mechanisms to improve athletic performance (Markovic and Mikulic, 2010). Plyometric training can be applied to nearly all sports (Haff and Triplett, 2015), and whenever we discuss jump training, we associate it with plyometric training (Kraemer et al., 2001). When we discuss jump training in this context, we mainly refer to plyometric training, although it should be noted that not all jump-based exercises fall into this category; some may involve additional resistance and relate more closely to strength training (Baker, 1996). Although this study specifically focuses on jump performance, it is important to note that plyometric training also contributes to improvements in other athletic movements such as sprinting, agility, and change of direction through enhanced neuromuscular adaptations and increased power output (Markovic and Mikulic, 2010).

Plyometric training is widely used to enhance physical performance in various sports activities involving sprinting, jumping, and changing direction (Kons et al., 2023). Studies have shown that athletes participating in plyometric training experience significant improvements in explosive and movement-related performance (de Villarreal et al., 2009; De Villarreal et al., 2010; Ramírez-Campillo et al., 2014; Asadi et al., 2016a). Effective jumping performance is crucial for success in sports involving frequent jumping and rapid changes of direction (Sattler et al., 2012). For instance, in volleyball, a good jump can enhance a player’s spiking, blocking, and serving abilities (Ziv and Lidor, 2010; Sheppard et al., 2007; Sheppard et al., 2009). In basketball, higher jumps benefit shooting and rebounding (Makaruk et al., 2020), while in soccer, the explosive power demonstrated in jumping is critical for heading the ball and other technical skills (Marcolin and Petrone, 2025). Moreover, research indicates that plyometric training can enhance neuromuscular function and coordination, thereby improving overall athletic performance (Hewett et al., 1996; Eraslan et al., 2021). It has also been shown to increase lower limb strength and muscle activation, providing a solid foundation for explosive movements (Deng et al., 2022; Ramirez-Campillo et al., 2021; Kubo et al., 2021; Guan et al., 2021).

Plyometric training has been proven to effectively improve athletes’ physical performance (Markovic, 2007; De Villarreal et al., 2010; Eraslan et al., 2021; Spurrs et al., 2003; Chen et al., 2023; Ramírez-delaCruz et al., 2022; Wang et al., 2023; Huang et al., 2023; Deng et al., 2023; Galay et al., 2021; Lum et al., 2023). Despite the numerous benefits of plyometric training, there is a lack of further investigation into the training performance of athletes and the application of different training methods compared to routine training. Previous studies often present varying results due to differences in research design, participant characteristics, and training protocols (Marczyk et al., 2010). Therefore, a systematic review and meta-analysis are needed to integrate these findings and provide a comprehensive understanding of the effects of plyometric training on jump performance. This systematic review and meta-analysis aim to evaluate the impact of plyometric training on different jump performances in athletes. This study aims not only to evaluate the absolute effectiveness of plyometric training on jump performance, but also to compare its effectiveness with routine training methods, thereby clarifying its relative advantages. By following the PRISMA guidelines (Sarkis-Onofre et al., 2021; Page et al., 2021a; Page et al., 2021b) and employing rigorous inclusion and exclusion criteria, this study seeks to provide a robust synthesis of the existing literature, thereby offering best practice guidance for athletes and coaches to optimize athletic performance.

## 2 Methods

### 2.1 Protocol and registration

This systematic review and meta-analysis adhered to the guidelines of the PRISMA statement (Page et al., 2021c), with the review protocol registered on Inplasy.com (DOI number: 10.37766/inplasy2024.6.0107).

### 2.2 Search strategy

Two reviewers (MS and XY) independently performed the search, identification, screening, and data extraction processes, with any discrepancies resolved by a third reviewer (QT). A comprehensive search was then conducted in electronic databases including PubMed, ERIC, Google Scholar, Web of Science, EBSCOhost, and Scopus to identify relevant studies. The search terms used were: (“plyometric exercise*” OR “plyometric training”) AND (“vertical jump” OR “vertical leap” OR “squat jump” OR “counter movement jump” OR “drop jump” OR “depth jump”). The search was confined to peer-reviewed articles published in English up to January 2025.

### 2.3 Inclusion criteria

A PICOS framework was utilized to evaluate the eligibility of the studies (McKenzie et al., 2019), with specific criteria detailed in Table 1.

**TABLE 1:** Inclusion criteria according to the PICOS conditions.

Items	Detailed inclusion criteria
Population	Healthy athletes
Intervention	All types of plyometric training (e.g., jump-based exercises)
Comparison	Two or more groups, with the control group on routine training
Outcome	Jump Performance in athletes
Study designs	RCT

### 2.4 Exclusion criteria

The exclusion criteria were as follows: (1) Studies that were not fully accessible; (2) studies not published in English; (3) Studies not focused on athletes’ jump performance; (4) Reviews, book reviews, cross-sectional studies, etc.; (5) studies that did not report numerical results.

### 2.5 Data extraction

Data extraction included information on athlete type, age, gender, height, weight, sample size, intervention description (type, intensity, duration, and frequency of the intervention), and study outcomes. This task was undertaken by one author (MS), with another author (XY) verifying the accuracy and completeness of the extracted data. Quality assessment was conducted by author (MS), with discrepancies resolved through consensus with (XY). Unresolved issues were referred to a third reviewer (QT) for adjudication.

### 2.6 Quality assessment

Following the guidelines provided on the Cochrane Training website, two reviewers (MS and XY) independently evaluated the risk of bias for each identified RCT using the revised Cochrane risk of bias (RoB) tool for randomized trials (RoB 2) (Higgins and Green, 2008). The Review Manager system was applied to assess and summarize the confidence in the evidence, adhering to the principles outlined in the GRADE handbook (Schünemann et al., 2020). This work was authored by two individuals (MS and XY). In case of any disagreements between the two authors, a third author (QT) was consulted.

### 2.7 Statistical analyses

All statistical analyses were conducted using the Review Manager software package. We extracted and analyzed the mean, standard deviation, and correlation coefficients for various outcome data. The change values were calculated as the difference between post-intervention and pre-intervention means. The standard deviations of the change scores were estimated using the formula:

Mean Change=Mean post−Mean pre

Standardized mean difference (SMD) was used as the effect size measure to account for variations in measurement scales across studies, allowing for a more consistent comparison of the intervention effects. For metrics included in this study with a sample size greater than or equal to three, forest plots and subgroup analyses were performed. The I^2^ statistic was used to assess heterogeneity among studies, with thresholds of 25%, 50%, and 75% representing low, moderate, and high levels, respectively. The choice between fixed-effects and random-effects models was based on the level of heterogeneity, with random-effects models applied when substantial heterogeneity was present (I^2^ > 50%) to account for between-study variability, while fixed-effects models were used for low heterogeneity scenarios (I^2^ ≤ 50%). All estimates were reported with their corresponding 95% confidence intervals (CI). The α level was set at 0.05 to indicate statistical significance.

## 3 Results

### 3.1 Study selection

**FIGURE 1:**
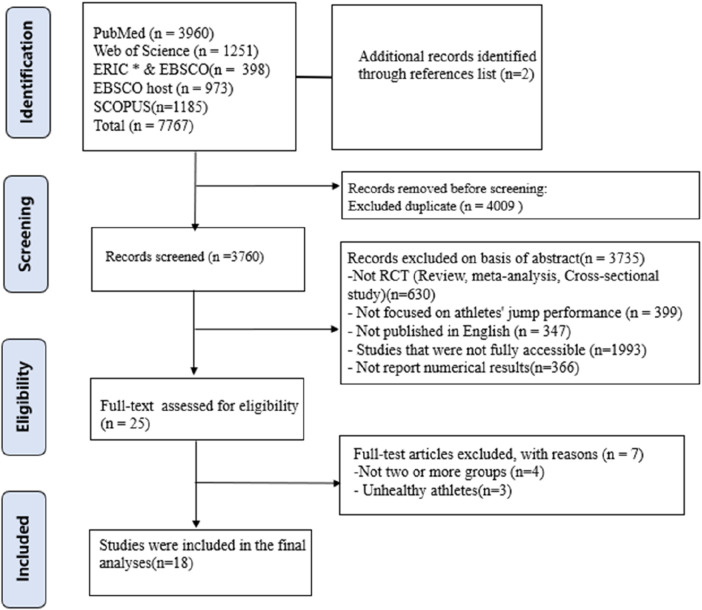
PRISMA flow diagram for study selection process.

In the comprehensive database search across PubMed, ERIC, Web of Science, EBSCOhost, and Scopus, 7767 articles were retrieved. Two more articles was added to the reference lists, totaling 7769 for initial review. Post-deduplication and exclusion of articles based on initial title and abstract analysis, the corpus was narrowed down to 3760 articles. These were independently evaluated on title and abstract criteria by researchers (MS and XY), from which 25 were deemed suitable for full-text examination. In the final selection stage, 7 articles were excluded as they did not conform to the strict inclusion and exclusion criteria, leaving 18 studies to be featured in this systematic review and meta-analysis, as detailed in Figure 1.

### 3.2 Methodological quality

**FIGURE 2:**
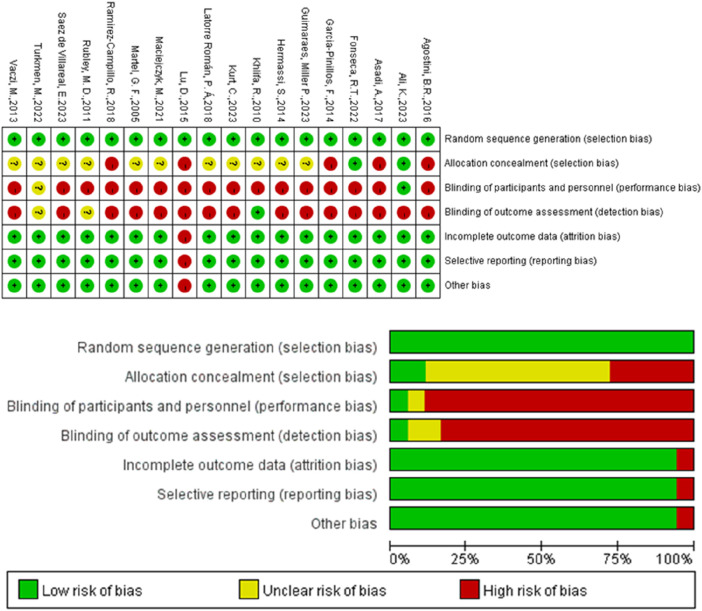
Risk of bias graph and summary.

In Figure 2, the quality of various studies is displayed. Colors indicate the likelihood of each type of bias occurring in the studies: green signifies a low risk of bias (RoB), yellow indicates an unclear RoB, and red represents a high RoB. This study found that in these 18 studies, the risks associated with random sequence generation (selection bias), incomplete outcome data (attrition bias), selective reporting (reporting bias), and other biases were relatively low. However, there was a notably higher unclear RoB in allocation concealment (selection bias), and a high risk of bias in blinding of participants and personnel (performance bias) as well as in blinding of outcome assessment (detection bias).

### 3.3 Study characteristics

In this systematic review and meta-analysis, the characteristics of the studies are delineated according to the PICO framework as follows.

#### 3.3.1 Population

The total sample size of this study was 573 participants. Four studies included female participants (Rubley et al., 2011; Guimarães et al., 2023; Martel et al., 2005; Agostini et al., 2017), and 14 studies included male participants (Maciejczyk et al., 2021; Ramirez-Campillo et al., 2018; Váczi et al., 2013; Saez de Villareal et al., 2023; Türkmen et al., 2022; Asadi et al., 2016b; Lu, 2025; Hermassi et al., 2014; Khlifa et al., 2010; Latorre Román et al., 2018; García-Pinillos et al., 2014; Kurt et al., 2023; Fonseca et al., 2022; Ahmed, 2015). The oldest participant in the studies was 24.16 ± 0.19 years old (Khlifa et al., 2010), and the youngest was 8.72 ± 0.97 years old (Latorre Román et al., 2018). The tallest participant was 192.58 ± 0.86 cm (Khlifa et al., 2010), and the shortest was 133 ± 7 cm (Latorre Román et al., 2018). The heaviest participant weighed 88.6 ± 1.1 kg (Hermassi et al., 2014), and the lightest was 30.56 ± 6.89 kg (Latorre Román et al., 2018). Among these studies, one study focused on gymnastics (Agostini et al., 2017), one on cricket (Ahmed, 2015), eight on soccer (Rubley et al., 2011; Ramirez-Campillo et al., 2018; Váczi et al., 2013; Türkmen et al., 2022; García-Pinillos et al., 2014; Kurt et al., 2023; Fonseca et al., 2022; Iacono et al., 2017), three on volleyball (Guimarães et al., 2023; Martel et al., 2005; Lu, 2025), three on baseball (Asadi et al., 2016b; Khlifa et al., 2010; Latorre Román et al., 2018), and three on handball (Saez de Villareal et al., 2023; Hermassi et al., 2014). A summary of these population characteristics is presented in Table 2.

**TABLE 2:** Characteristics of the study population.

Characteristic	Range/Value
Total participants	573
Age (years)	8.72 ± 0.97–24.16 ± 0.19
Height (cm)	133 ± 7–192.58 ± 0.86
Weight (kg)	30.56 ± 6.89–88.6 ± 1.1
Sport	Gymnastics, Cricket, Soccer, Volleyball, Baseball, Handball

#### 3.3.2 Intervention

In this systematic review and meta-analysis, the duration of interventions ranged from 4 to 14 weeks. Three studies had a 4-week intervention (Guimarães et al., 2023; Maciejczyk et al., 2021; Ahmed, 2015), one study had a 5-week intervention (Martel et al., 2005), three studies had a 6-week intervention (Váczi et al., 2013; Kurt et al., 2023; Fonseca et al., 2022), six studies had an 8-week intervention (Ramirez-Campillo et al., 2018; Saez de Villareal et al., 2023; Türkmen et al., 2022; Asadi et al., 2016b; Lu, 2025; Hermassi et al., 2014), two studies had a 10-week intervention (Khlifa et al., 2010; Latorre Román et al., 2018), one study had a 12-week intervention (García-Pinillos et al., 2014), and one study had a 14-week intervention (Rubley et al., 2011).

The frequency of interventions ranged from 1 to 7 times per week. One study conducted the intervention once a week (Rubley et al., 2011), nine studies conducted it twice a week (Maciejczyk et al., 2021; Ramirez-Campillo et al., 2018; Váczi et al., 2013; Hermassi et al., 2014; Latorre Román et al., 2018; García-Pinillos et al., 2014; Kurt et al., 2023; Fonseca et al., 2022; Ahmed, 2015), five studies conducted it three times a week (Agostini et al., 2017; Saez de Villareal et al., 2023; Türkmen et al., 2022; Asadi et al., 2016b; Lu, 2025), one study conducted it five times a week (Martel et al., 2005), one study conducted it six times a week (Khlifa et al., 2010), and one study conducted it seven times a week (Guimarães et al., 2023).

In terms of the duration of each intervention session, one study had interventions lasting 10–29 min per week (Latorre Román et al., 2018), one study had interventions lasting 40 min per week (Lu, 2025), two studies had interventions lasting 90 min each session (Khlifa et al., 2010; Kurt et al., 2023), one study had interventions lasting 100 min each session (Martel et al., 2005), and 13 studies did not specify the duration of each intervention session (Rubley et al., 2011; Guimarães et al., 2023; Agostini et al., 2017; Maciejczyk et al., 2021; Ramirez-Campillo et al., 2018; Váczi et al., 2013; Saez de Villareal et al., 2023; Türkmen et al., 2022; Asadi et al., 2016b; Hermassi et al., 2014; García-Pinillos et al., 2014; Fonseca et al., 2022; Ahmed, 2015).

#### 3.3.3 Comparison

One study designed four test groups (Agostini et al., 2017), two studies designed two test groups (Rubley et al., 2011; Kurt et al., 2023), and the remaining studies each designed one test group (Rubley et al., 2011; Guimarães et al., 2023; Agostini et al., 2017; Maciejczyk et al., 2021; Ramirez-Campillo et al., 2018; Váczi et al., 2013; Saez de Villareal et al., 2023; Türkmen et al., 2022; Asadi et al., 2016b; Hermassi et al., 2014; García-Pinillos et al., 2014; Fonseca et al., 2022; Ahmed, 2015).

#### 3.3.4 Outcome

In this systematic review and meta-analysis, seven studies focused on vertical jump (VJ) (Rubley et al., 2011; Agostini et al., 2017; Váczi et al., 2013; Türkmen et al., 2022; Asadi et al., 2016b; Lu, 2025; Kurt et al., 2023), nine studies focused on counter movement jump (CMJ) (Guimarães et al., 2023; Martel et al., 2005; Maciejczyk et al., 2021; Ramirez-Campillo et al., 2018; Saez de Villareal et al., 2023; Khlifa et al., 2010; Latorre Román et al., 2018; García-Pinillos et al., 2014; Fonseca et al., 2022), three studies focused on squat jump (SJ) (Maciejczyk et al., 2021; Ramirez-Campillo et al., 2018; Hermassi et al., 2014), and one study focused on standing vertical jump (SVJ) (Ahmed, 2015). Specific details are shown in Table 3.

**TABLE 3:** Characteristics.

Author, year	Population					Intervention and comparison					Outcome			
	Athletes	Age (years)	Gender	Height (cm)	Weight (kg)	Type	Sample size (n)	Duration (weeks)	Frequency (days/Week)	Session (min)	CMJ (%)	SJ (%)	SVJ (%)	VJ (%)
Agostini et al. (2017)	Gymnastics	C: 15.2 ± 1.5 T: 15.4 ± 1.2	F	C: 165 ± 7 T: 169 ± 2	-	C: RT T: PT	30 (15/15)	12	3					C: 9.7↑* T: 8.4↑*
		C: 15.2 ± 1.5 T: 15.4 ± 1.2	F	C: 165 ± 7 T: 169 ± 2	-	C: RT T: PT	30 (15/15)	24	3					C: 18.1↑* T: 16↑*
		C: 15.2 ± 1.5 T: 15.4 ± 1.2	F	C: 165 ± 7 T: 169 ± 2	-	C: RT T: PT	30 (15/15)	36	3					C: 23.5↑* T: 28.7↑*
		C: 15.2 ± 1.5 T: 15.4 ± 1.2	F	C: 165 ± 7 T: 169 ± 2	-	C: RT T: PT	30 (15/15)	48	3					C: 28.4↑* T: 42.3↑*
Ali et al. (2023)	Cricket	C: 19.7 ± 1.3 T: 19.5 ± 1.6	M	C: 176.7 ± 6.1 T: 174.9 ± 8.4	C: 64.4 ± 10.2 T: 62.9 ± 11	C: RT T: PT	28 (14/14)	4	2				C: 0.6↓ T: 11.8↑	
Fonseca et al. (2022)	Soccer	C: 17.3 ± 0.5 T: 17.3 ± 0.7	M	C: 174.6 ± 7.9 T: 175.9 ± 5.8	C: 68.3 ± 8 T: 65.4 ± 6.3	C: RT T: PT	17 (9/8)	6	2		C: 0.7↑ T: 7.6↑			
Martelet al. (2005)	Volleyball	C: 15.5 ± 1.52 T: 15.4 ± 1.32	F	C: 169.11 ± 5.88 T: 170.76 ± 4.81	C: 61.31 ± 7.61 T: 60.76 ± 8.43	C: RT T: PT	54 (27/27)	5	5	100	C: 0.8↑ T: 9.4↑*			
Guimaraes et al. (2023)	Volleyball	C: 19.5 ± 3.73 T: 19.5 ± 3.73	F	C: 182 ± 2.1 T: 180 ± 3	C: 66.9 ± 6.35 T: 67.7 ± 4.32	C: RT T: PT	17 (9/8)	4	7		C: 3.8↑ T: 9.5↑			
Rubley et al. (2011)	Soccer	C: 13.4 ± 0.5 T: 13.4 ± 0.5	F	C: 162.5 ± 5.67 T: 162.5 ± 5.67	C: 50.84 ± 5.1 T: 50.84 ± 5.1	C: RT T: PT	14 (7/7)	7	1					C: 7.6↓ T: 8.3↑*
		C:13.4 ± 0.5 T:13.4 ± 0.5	F	C: 162.5 ± 5.67 T: 162.5 ± 5.67	C: 50.84 ± 5.1 T: 50.84 ± 5.1	C: RT T: PT	14 (7/7)	14	1					C: 5.4↓ T: 18.7↑
Kurt et al. (2023)	Soccer	C: 12.09 ± 0.89 T: 12.09 ± 0.89	M	C: 155.01 ± 9.97 T: 155.01 ± 9.97	C: 44.58 ± 8.04 T: 44.58 ± 8.04	C: RT T: PT	23 (12/11)	6	2	90				C: 7.9↓ T: 0.7↓
		C: 12.09 ± 0.89 T: 12.09 ± 0.89	M	C: 155.01 ± 9.97 T: 155.01 ± 9.97	C: 44.58 ± 8.04 T: 44.58 ± 8.04	C: RT T: PT	23 (12/11)	6	2	90				C: 7.9↓ T: 0
Garcia-Pinillos et al (2014)	Soccer	C: 16.38 ± 1.5 T: 15.47 ± 1.28	M	C: 169 ± 0.06 T: 172 ± 0.05	C: 61.53 ± 9.46 T: 68.32 ± 11.18	C: RT T: PT	30 (17/13)	12	2		C: 2.2↑ T: 7.1↑*			
Latorre Román et al. (2018)	Baseball	C: 8.72 ± 0.97 T: 8.72 ± 0.97	M	C: 133 ± 7 T: 133 ± 7	C: 30.56 ± 6.89 T: 30.56 ± 6.89	C: RT T: PT	58 (30/28)	10	2	10–29	C: 6↑* T: 14.1↑*			
Khlifa et al. (2010)	Baseball	C: 24.16 ± 0.19 T: 23.57 ± 0.34	M	C: 192.58 ± 0.86 T: 191.73 ± 0.52	C: 82.61 ± 0.79 T: 81.72 ± 0.5	C: RT T: PT	18 (9/9)	10	6	90	C: 1.8↑* T: 7↑*			
Hermassi et al., 2014	Handball	C: 20.1 ± 0.2 T: 20.1 ± 0.3	M	C: 190 ± 2 T: 189 ± 3	C: 88.6 ± 1.1 T: 85.8 ± 3.1	C: RT T: PT	24 (14/10)	8	2			C: 5.1↑* T: 7.1↑*		
Lu (2015)	Volleyball	C: 17.5 ± 2.2 T: 17.5 ± 2.2	M	C: 178.3 ± 6.56 T: 178.3 ± 6.56	C: 67.6 ± 5.87 T: 67.6 ± 5.87	C: RT T: PT	20 (10/10)	8	3	40				C: 0 T: 1.5↑
Asadi et al. (2016b)	Baseball	C: 18.5 ± 0.8 T: 18.5 ± 0.8	M	C: 186.1 ± 5.6 T: 186.1 ± 5.6	C: 78.4 ± 7.6 T: 78.4 ± 7.6	C: RT T: PT	16 (8/8)	8	3					C: 0.2↑ T: 14.3↑*
Turkmen et al. (2022)	Soccer	C: 17.7 ± 0.5 T: 17.2 ± 0.4	M	C: 179.7 ± 9.8 T: 175.8 ± 5.1	C: 70.7 ± 12.1 T: 61.4 ± 4.3	C: RT T: PT	16 (8/8)	8	3					C: 2.5↑* T: 13.5↑*
Saez de Villareal et al. (2023)	Handball	C: 20.6 ± 1.6 T: 19.8 ± 2.2	M	C: 180.2 ± 2.8 T: 178.3 ± 4.3	C: 81.2 ± 5.2 T: 79.1 ± 8.3	C: RT T: PT	24 (12/12)	8	3		C: 1.6↑ T: 7.1↑			
Váczi et al. (2013)	Soccer	C: 22.7 ± 1.4 T: 21.9 ± 1.7	M	C: 180.6 ± 3.7 T: 180.1 ± 4	C: 78.6 ± 3.1 T: 75.9 ± 2.7	C: RT T: PT	24 (12/12)	6	2					C: 0.2↓ T: 8.9↑*
Ramirez-Campillo et al. (2018)	Soccer	C: 17.6 ± 0.5 T: 17.3 ± 1.1	M	C: 174.9 ± 5.3 T: 177.1 ± 5.9	C: 68.3 ± 3.6 T: 64.9 ± 5.5	C: PT T: PT	18 (9/9)	8	2		C: 8.1↑* T: 5.3↑	C: 5.7↑* T: 5.7↑		
Maciejczyk et al. (2021)	Soccer	C: 18.2 ± 1.8 T: 21 ± 3	M	C: 161.7 ± 4.3 T: 164.5 ± 6.91	C: 55 ± 5.39 T: 61.3 ± 13.86	C: RT T: PT	15 (7/8)	4	2		C: 1.8↑ T: 6.5↑*	C: 3.9↑ T: 9.1↑*		

V,vertical jump; SVJ, stationary vertical jump; CMJ, counter movement jump; SJ, squat jump; C, control group; T, treatment group; M, male; F, female; PT, plyometric training; RT, routine training; ↑*, indicates significant increase; ↑ indicates increase.

## 4 Meta-analysis

Meta-analysis is used to summarize and integrate the results of multiple studies. Therefore, in this systematic review and meta-analysis, we limited the meta-analysis to indicators that include data from three or more studies. Ultimately, this systematic review conducted a meta-analysis on the indicators of counter movement jump, squat jump, and vertical jump. The standing vertical jump indicator was excluded from the meta-analysis due to insufficient data.

### 4.1 Counter movement jump

**FIGURE 3:**
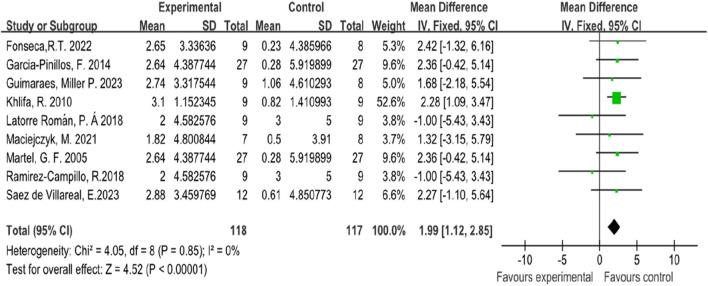
Forest map of influence of plyometric training on counter movement jump.

In this systematic review and meta-analysis, nine studies focused on the indicators of athletes’ counter movement jump performance (Guimarães et al., 2023; Martel et al., 2005; Maciejczyk et al., 2021; Ramirez-Campillo et al., 2018; Saez de Villareal et al., 2023; Khlifa et al., 2010; Latorre Román et al., 2018; García-Pinillos et al., 2014; Fonseca et al., 2022). Among these studies, two involved female subjects (Guimarães et al., 2023; Martel et al., 2005), while seven involved male subjects (Maciejczyk et al., 2021; Ramirez-Campillo et al., 2018; Saez de Villareal et al., 2023; Khlifa et al., 2010; Latorre Román et al., 2018; García-Pinillos et al., 2014; Fonseca et al., 2022). The forest plot of the meta-analysis showed that plyometric training, with an average SMD of 1.99 (I^2^ = 0%, Chi^2^ = 4.05, df = 8, P < 0.001), significantly improved the counter movement jump performance of athletes compared to routine training. This indicates that plyometric training has a significant effect on athletes’ counter movement jump performance, and there is minimal heterogeneity among these nine studies, as illustrated in Figure 3.

### 4.2 Squat jump

**FIGURE 4:**
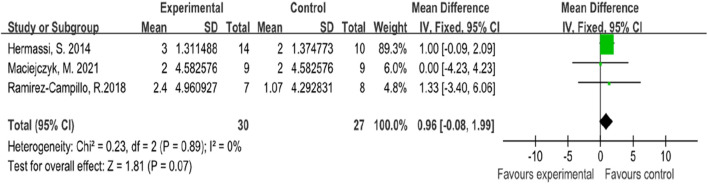
Forest map of influence of plyometric training on squat jump.

In this systematic review and meta-analysis, three studies focused on the indicators of athletes’ squat jump performance, all of which involved male subjects (Maciejczyk et al., 2021; Ramirez-Campillo et al., 2018; Hermassi et al., 2014). The forest plot of the meta-analysis showed an average SMD of 0.96 (I^2^ = 0%, Chi^2^ = 0.23, df = 2, P = 0.07), indicating that there is no significant difference between plyometric training and routine training in improving athletes’ squat jump performance. The heterogeneity among the three studies is minimal, as illustrated in Figure 4.

### 4.3 Vertical jump

**FIGURE 5:**
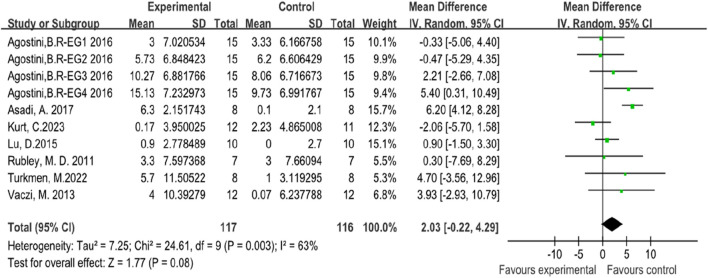
Forest map of influence of plyometric training on vertical jump.

In this systematic review and meta-analysis, seven studies focused on the indicators of athletes’ vertical jump performance (Rubley et al., 2011; Agostini et al., 2017; Váczi et al., 2013; Türkmen et al., 2022; Asadi et al., 2016b; Lu, 2025; Kurt et al., 2023). Among these studies, two involved female subjects (Rubley et al., 2011; Agostini et al., 2017), while five involved male subjects (Váczi et al., 2013; Türkmen et al., 2022; Asadi et al., 2016b; Lu, 2025; Kurt et al., 2023). The forest plot of the meta-analysis showed an average SMD of 2.90347 (I^2^ = 63%, Chi^2^ = 24.61, df = 9, P = 0.08), indicating that there is no significant difference between plyometric training and routine training in improving athletes’ vertical jump performance. The heterogeneity among the seven studies is relatively high, as illustrated in Figure 5.

**FIGURE 6:**
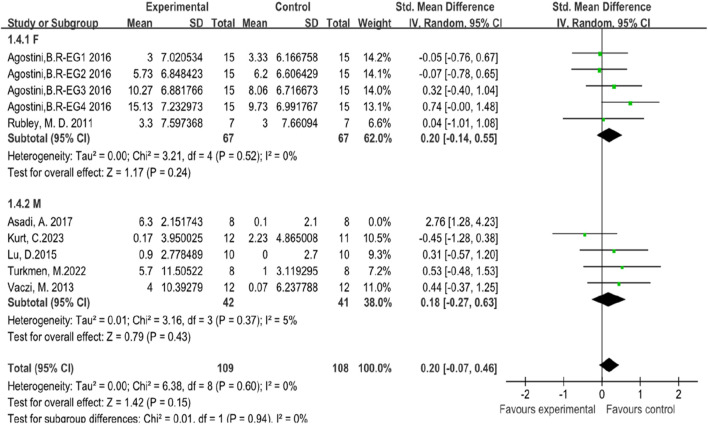
Subgroup analysis of vertical jump performance.

Due to the high heterogeneity among the seven studies, a subgroup analysis was conducted based on gender. The subgroup analysis results showed the following: For the two studies involving female subjects, the results were (I^2^ = 0%, Chi^2^ = 3.21, df = 4, P = 0.24), indicating that there was no significant difference between plyometric training and routine training in improving athletes’ vertical jump performance, with low heterogeneity among the studies. For the five studies involving male subjects, the results were (I^2^ = 71%, Chi^2^ = 17.62, df = 9, P = 0.07), indicating that there was no significant difference between plyometric training and routine training in improving athletes’ vertical jump performance, but with high heterogeneity among the studies. Heterogeneity testing was conducted by sequentially excluding studies, and it was found that excluding the study by Asadi, A. (2016) reduced the I^2^ from 71% to 5%, thus identifying the source of heterogeneity (Asadi et al., 2016b). The detailed results are illustrated in Figure 6.

## 5 Discussion

This systematic review and meta-analysis demonstrate that plyometric training significantly enhances athletes’ counter movement jump performance compared to routine training, while it does not show superior effects on squat jump and vertical jump performance.

### 5.1 Counter movement jump

The meta-analysis results for counter movement jump performance indicated a significant improvement with plyometric training compared to routine training. The standardized mean difference was 1.99 (Chi^2^ = 4.05, P < 0.001), suggesting that plyometric training is more effective in enhancing counter movement jump performance among athletes. The low heterogeneity (I^2^ = 0%) among the studies further supports the robustness of this finding. This result aligns with previous research that highlights the benefits of plyometric training in improving explosive power and jump performance in athletes (Markovic, 2007; De Villarreal et al., 2009).

The superior improvements in CMJ performance can be attributed to specific neuromuscular and biomechanical adaptations induced by plyometric training (Arabatzi et al., 2010). Neuromuscularly, it increases the recruitment of high-threshold motor units and enhances muscle spindle sensitivity, enabling faster reflex activation and greater force production (Lee, 2023). At the muscle–tendon level, adaptations such as increased tendon stiffness and improved elastic energy reutilization allow more efficient use of the stretch–shortening cycle (van Ingen Schenau et al., 1997). Furthermore, improved hip–knee–ankle coordination reduces transition time between eccentric and concentric phases, thereby optimizing power output (Huang et al., 2025). These mechanisms collectively explain the greater effectiveness of plyometric training compared to routine training in enhancing CMJ performance. In the meta-analysis of counter movement jump performance, the studies included soccer players (Fonseca et al., 2022), volleyball players (Guimarães et al., 2023; Martel et al., 2005), soccer players (García-Pinillos et al., 2014), basketball players (Khlifa et al., 2010; Latorre Román et al., 2018), handball players (Saez de Villareal et al., 2023), and soccer players (Maciejczyk et al., 2021; Ramirez-Campillo et al., 2018). Counter movement jump performance is undoubtedly important for these athletes, and this study confirms the effectiveness of plyometric training compared to routine training in improving athletes’ counter movement jump performance.

In a counter movement jump, the quadriceps, gluteus maximus, and gastrocnemius muscles eccentrically contract during the eccentric phase to store elastic energy, rapidly transition to the concentric phase to minimize energy loss, and then concentrically contract to propel the body upward. This stretch-shortening cycle enhances muscle-tendon elasticity and neuromuscular efficiency, resulting in higher jumps (Komi, 2000; Markovic, 2007; de Villarreal et al., 2009). This cycle enhances the elastic properties of the muscle-tendon complex and increases neuromuscular efficiency, leading to improved explosive strength and power (Komi, 2000), thus making it more effective than Routine training. Numerous studies have confirmed that plyometric training can improve athletes’ use of the stretch-shortening cycle (Kyröläinen et al., 2005; McBride et al., 2008; Myer et al., 2006).

### 5.2 Squat jump

For squat jump performance, the meta-analysis did not show a significant difference between plyometric training and routine training. The standardized mean difference was 0.96 (Chi^2^ = 0.23, P = 0.07), indicating that both training methods are similarly effective in improving squat jump performance among athletes. The low heterogeneity (I^2^ = 0%) suggests that the findings are consistent across the included studies (Huedo-Medina et al., 2006).

The squat jump mainly involves concentric muscle actions without the preceding eccentric phase, which explains why routine training can be as effective as plyometric training. Routine training enhances muscle strength through hypertrophy and neural adaptations, which are critical for the force production needed in the squat jump (Suchomel et al., 2016; Faigenbaum et al., 2007). During the concentric phase, muscle fibers, particularly type II fibers, are activated to generate maximal force (Stone et al., 1981). Routine training typically involves high-intensity, low-repetition exercises that target these fast-twitch fibers, leading to increased muscle cross-sectional area and enhanced neuromuscular efficiency (HÄkkinen et al., 1985).The absence of the eccentric phase in the squat jump means that the stretch-shortening cycle, which is a key component of plyometric training, does not play a significant role (Turner and Jeffreys, 2010). Instead, the focus is on the concentric phase, where muscle strength and power are paramount. The ability to generate high force in a short time is essential, and routine training improves this ability through increased motor unit recruitment and firing rates (Aagaard et al., 2002).

Moreover, routine training typically involves exercises that increase muscle mass and improve the neuromuscular connection, leading to better recruitment of muscle fibers during the concentric phase (Weisbrod et al., 2001; Henneman et al., 1965). This can result in similar improvements in squat jump performance as those achieved through plyometric training, which relies more on explosive power and rapid movements (Markovic, 2007). Therefore, athletes and coaches can consider incorporating either training method based on convenience and specific training goals, as both have been shown to be effective in enhancing squat jump performance.

### 5.3 Vertical jump

The analysis of vertical jump performance revealed no significant difference between plyometric training and routine training, with a standardized mean difference of 2.90 (I^2^ = 63%, P = 0.08). The high heterogeneity (I^2^ = 63%) indicates substantial variability among the studies. To address this issue, a subgroup analysis based on gender was conducted. For female subjects, there was no significant difference between plyometric training and routine training (Chi^2^ = 3.21, P = 0.24), with low heterogeneity among the studies (I^2^ = 0%). For male subjects, there was no significant difference between plyometric training and routine training (Chi^2^ = 17.62, P = 0.07), but the heterogeneity was high (I^2^ = 71%). Excluding the study by Asadi (2016) significantly reduced the heterogeneity to 5%, indicating that this study was a major source of variability (Asadi et al., 2016b). This may be due to differences in participant characteristics and training protocols used in that study compared with the others.

The biological mechanism behind vertical jump improvement involves both the stretch-shortening cycle and maximal muscle power output. Plyometric training enhances the efficiency of the SSC, contributing to improved jump height (Komi, 2000). However, the varying results among male subjects suggest that individual differences, training background, and methodological differences in the studies may influence the outcomes (Miller et al., 2006; Rhea et al., 2009). Further research is needed to explore the specific factors contributing to this variability and to establish clearer guidelines for training interventions.

### 5.4 Narrative synthesis and practical contributions

In addition to the quantitative meta-analysis, this systematic review also provides a narrative synthesis, highlighting variations in participant characteristics (e.g., sport type and gender), intervention duration, training frequency, and outcome measurement methods across the included studies. Although relevant physiological mechanisms and previous studies support the theoretical foundation for plyometric training’s effectiveness, the relatively low heterogeneity observed in the counter movement jump and squat jump outcomes adds methodological strength to our findings. Importantly, unlike many previous systematic reviews that focused solely on the absolute effectiveness of plyometric training, this study directly compares plyometric training with routine training. This comparative perspective offers more practical value by clarifying the relative advantages of plyometric interventions in real-world athletic settings. Therefore, our findings provide coaches and practitioners with more targeted evidence to guide training program design.

## 6 Limitations

### 6.1 Study heterogeneity

High heterogeneity was observed in the vertical jump performance studies, particularly among male subjects. This variability suggests differences in study design, participant characteristics, and training protocols.

### 6.2 Limited female representation

The number of studies involving female subjects was relatively small, which may affect the generalizability of the results to female athletes.

### 6.3 Publication bias

As with any meta-analysis, there is a risk of publication bias, where studies with significant findings are more likely to be published than those with non-significant results.

## 7 Conclusion

This study demonstrates that plyometric training significantly enhances counter movement jump performance, while showing no superior effects on squat jump and vertical jump performance compared with routine training. These findings highlight the specificity of plyometric adaptations and suggest that practitioners should prioritize such training when aiming to improve explosive jump performance. Future research should investigate optimal training protocols and contextual factors that may modulate its effectiveness across different jump types.

## Data Availability

The original contributions presented in the study are included in the article/supplementary material, further inquiries can be directed to the corresponding author.
